# Psoriasis, Psoriatic Arthritis, and Thyroid Autoimmunity

**DOI:** 10.3389/fendo.2017.00139

**Published:** 2017-06-19

**Authors:** Ilaria Ruffilli, Francesca Ragusa, Salvatore Benvenga, Roberto Vita, Alessandro Antonelli, Poupak Fallahi, Silvia Martina Ferrari

**Affiliations:** ^1^Department of Clinical and Experimental Medicine, University of Pisa, Pisa, Italy; ^2^Department of Clinical and Experimental Medicine, University of Messina School of Medicine, Messina, Italy; ^3^Master Program of Childhood, Adolescence and Women’s Endocrine Health, University of Messina School of Medicine, Messina, Italy; ^4^Interdepartmental Program of Molecular & Clinical Endocrinology, and Women’s Endocrine Health, University Hospital, Messina, Italy

**Keywords:** psoriasis, psoriatic arthritis, autoimmune thyroiditis, hypothyroidism, CXCL10, AbTPO, anti-thyroglobulin antibodies

## Abstract

Psoriasis (PsO) is a chronic relapsing/remitting autoimmune skin disease, associated with an increased risk of other autoimmune disorders. Psoriatic arthritis (PsA) is a chronic inflammatory arthritis occurring approximately in 30% of PsO patients. Sporadic cases of association between PsO and autoimmune thyroid disorders (AITDs) have been reported. However, two different recent studies did not find any association between them. In patients with PsO and PsA, an association with AITD has been shown by most of the studies in adults, but not in the juvenile form. In PsA women and men, thyroid autoimmunity [positive antithyroid peroxidase (AbTPO) antibodies, hypoechoic thyroid pattern] and subclinical hypothyroidism were more prevalent than in the general population. An association has been shown also in patients with PsO, arthritis, and inflammatory bowel disease, who have more frequently AITD. A Th1 immune predominance has been shown in early PsO, and PsA, with high serum CXCL10 (Th1 prototype chemokine), overall in the presence of autoimmune thyroiditis. This Th1 immune predominance might be the immunopathogenetic base of the association of these disorders. A raised incidence of new cases of hypothyroidism, thyroid dysfunction, positive AbTPO, and appearance of a hypoechoic thyroid pattern in PsA patients, especially in women, has been shown recently, suggesting to evaluate AbTPO levels, thyroid function, and thyroid ultrasound, especially in PsA women. Thyroid function follow-up and suitable treatments should be performed regularly in PsA female patients at high risk (thyroid-stimulating hormone within the normal range but at the higher limit, positive AbTPO, hypoechoic, and small thyroid).

## Introduction

Psoriasis (PsO) (1) affects about 2–4% of the population (2); it is a chronic relapsing/remitting autoimmune skin disease (1) and presents with itchy red, scaly patches, papules, and plaques, with different severity, from localized patches to general body coverage. PsO is classified in five types: plaque, guttate, inverse, pustular, and erythrodermic (3). These lesions are usually evident on the skin of elbows and knees, and also on scalp, palms of hands, and soles of feet. Psoriatic nail dystrophy is usually present in fingernails and toenails and can be an isolated sign. A genetic predisposition is very important in the pathogenesis of PsO; however, environmental factors can activate the disease (1).

The skin epidermal layer grows rapidly in PsO (4), determining an abnormal production and an excess of skin cells (5), that are replaced in 3–5 days in PsO (while commonly every 28–30 days) (6). These events are probably induced by the premature keratinocytes maturation, induced by the inflammatory cascade in the dermis (7). The immune competent cells go from dermis to epidermis and release different inflammatory cytokines [interleukin (IL)-1β, interferon (IFN)-γ, tumor necrosis factor-α, chemokine (C-X-C motif) ligand (CXCL)10, IL-6, IL-22] (8). In PsO, DNA can stimulate the dendritic cells, to produce IFN-α. The secretion of such inflammatory cytokines leads to stimulate the proliferation of keratinocytes (8).

Psoriatic arthritis (PsA) is a chronic inflammatory arthritis that has a variable clinical presentation and occurs approximately in 30% of PsO patients (7, 9, 10). PsA typically affects the joints of the fingers and toes, and it is characterized by a painful inflammation of the joints and surrounding connective tissue. This process results in a sausage-shaped swelling of the fingers and toes called dactylitis (9). PsA can also affect the hips, spine (spondylitis), knees, and sacroiliac joint (sacroiliitis), and any other joint (11). Dermatologic manifestations of PsO appear before the manifestations of arthritis in about 75% of patients (10).

Psoriasis is associated with an increased risk of other autoimmune disorders like ulcerative colitis, Crohn’s disease, and autoimmune thyroiditis (AT) too (12).

Here, we review the scientific literature about PsO, PsA, and the possible association with autoimmune thyroid disorders (AITDs).

## PsO, PsA, and AITD

Few case reports initially reported an association of PsO and Hashimoto’s thyroiditis (13, 14) (Table 1). A first systematic study by Bianchi et al. (15) evaluated thyroid volume and function and the prevalence of anti-microsome and anti-thyroglobulin antibodies (AbTg) in 42 patients with PsA, versus 52 normal subjects, as controls. The average thyroid volume, measured at ultrasounds, was increased in comparison to controls. Patients with PsA had a raised prevalence of anti-microsome antibodies; thyroid involvement was confined to patients with active disease. These results suggested a significant thyroid involvement in PsA patients. However, the study was basically a retrospective study, and it did not specify the selection criteria of the patients (15).

**Table 1:** Prevalence of thyroid autoimmunity in psoriasis (PsO), or psoriatic arthritis (PsA) patients, versus controls, in the studies that have an internal control group.

Reference	PsO patients (*n*)	Autoimmune thyroid disorder (AITD)% in PsO patients	Controls (*n*)	AITD% in controls	*P*
Antonelli et al. (12)	80 with PsA	12/36 of F patients (33%); 11/44 of M patients (25%)	112 patients with rheumatoid arthritis; 400 control subjects	33/180 of F controls (18%); 10/220 of M controls (5%)	0.0001
Bianchi et al. (15)	42 with PsA	AbTg prevalence 5%; anti-microsome antibodies prevalence 14%	52	AbTg prevalence 3%; anti-microsome antibodies prevalence 0%	<0.05
Gul et al. (16)	105	6 patients had increased AbTPO (6%); 8 patients had increased AbTg (8%); 3 patients had both increased AbTPO and AbTg (3%)	96 with tinea pedis	AbTPO levels were increased in 6 subjects (6%), AbTg levels were increased in 11 subjects (11%) and both of them were increased in 6 subjects (6%)	NS
Vassilatou et al. (17)	114 (30 of them with PsA)	Prevalence of autoimmune thyroiditis (AT) 20.2%	286	Prevalence of AT 19.6%	NS
Fallahi et al. (18)	97 with PsA	34% thyroid autoimmunity	97	15% thyroid autoimmunity	0.002

*AbTg, anti-thyroglobulin antibodies; AbTPO, anti-thyroperoxidase antibodies; F, females; M, males; NS, not significant*.

A second study evaluated the prevalence of thyroid disorders in PsA patients, conducting a complete thyroid work-out in 80 PsA patients, versus control subjects extracted (1:5) from the general population (matched by age and gender), and 112 patients with rheumatoid arthrtitis (RA) (with similar iodine intake). PsA women had significantly more frequently a hypoechoic thyroid pattern, antithyroid peroxidase antibodies (AbTPO), and subclinical hypothyroidism than control women, with a frequency comparable to that in RA patients (hypoechoic thyroid 31, 16, and 36%; positive AbTPO titer 28, 12, and 31%; subclinical hypothyroidism 25, 8, and 12%, respectively). PsA and RA men showed more frequently hypoechoic thyroid pattern and positive AbTPO than control subjects (hypoechoic thyroid 16, 10, and 3%; positive AbTPO titer 14, 5, and 2%, respectively). PsA patients with subclinical hypothyroidism had a longer disease duration (years; 19 ± 15 versus 11 ± 8, *p* = 0.03) and polyarticular involvement (*p* ≤ 0.05) than euthyroid PsA patients. Therefore, a significantly higher prevalence of thyroid autoimmunity (positive AbTPO, hypoechoic thyroid pattern) in PsA men and women, and of subclinical hypothyroidism in PsA women, than in the general population were evidenced (12). Conversely, a subsequent study investigated the frequency of rheumatic diseases in 65 patients (56 F, 9 M), suffering from AITD; antinuclear antibody and rheumatoid factor levels were also measured. Various rheumatic disorders were detected in 40 (62%) of patients with AITD: the most frequent were fibromyalgia, osteoarthritis, keratoconjunctivitis sicca, and xerostomia. Autoimmune diseases were detected in 10 patients with AITD, and among them also PsO and PsA (19). A further study (20) evaluated the frequency of AITD in 80 children with juvenile idiopathic arthritis (JIA) (27 oligoarticular, 26 polyarticular, 17 enthesitis-related, 6 systemic, and 4 PsA), versus 81 healthy control subjects, matched by age and gender. AITD were found in four patients in the JIA group (5%). No significant difference between the study and control groups was observed in the frequency of circulating antithyroid antibodies, or AT, suggesting that in JIA there is no association with AITD (20).

A further study evaluated thyroid autoimmunity in 105 patients with PsO (without PsA), versus 96 sex- and age-matching controls (with tinea pedis). The levels of free thyroxine (FT4) resulted significantly increased in the PsO group; however, AbTPO and AbTg levels were not significantly different between the two groups. The study showed that the serum FT4 levels can increase in psoriatic patients. However, thyroid-stimulating hormone (TSH), FT4, or FT3 were not reported. Furthermore, an increase of FT4 should be related to TSH decreased levels, however any correlation was reported (16).

The association between PsO and inflammatory bowel disease (IBD) has been previously reported, even if potential associated comorbidities are not clear. A study (21) examined comorbidities in 146 patients diagnosed with both PsO and IBD, in comparison with those diagnosed with PsO-only (146, matched by gender, ethnicity, and age). Patients with both PsO and IBD (versus PsO-only) had significantly higher rates of hepatitis (6.2 versus 0.7%), AT (6.8 versus 2.1%), and diabetes (26.77 versus 11.0%), and 60 (41.1%) were diagnosed with seronegative arthritis, suggesting that patients with both PsO and IBD have more frequently AITD and arthritis (21).

The prevalence of 12 autoimmune diseases was also evaluated in 25,885 people extracted from the general population in Sardinia (22). A high prevalence was observed for RA, ulcerative colitis, Crohn’s disease, type 1 diabetes, systemic lupus erythematosus, celiac disease, myasthenia gravis, systemic sclerosis, multiple sclerosis, Sjogren’s syndrome, PsO/PsA (939 cases), and AT (2,619 cases). The statistical analysis of the comorbidity of autoimmune diseases shows that the number of people with more than one autoimmune disease was significantly higher than the expected number, both in women and men (22).

Another study (17) evaluated prospectively 114 PsO patients with disease duration of 5–38 years, 30 of them with PsA, in comparison with 286 age- and body mass index-matched subjects. No difference in the prevalence of AT between PsO patients and controls (20.2 versus 19.6%) was present. The prevalence of AT in male and female PsO patients was similar (9.6 and 10.5%, respectively) unlike the increased, as expected, prevalence in female versus male controls (14.7 versus 4.9%). Detected cases with hypothyroidism due to AT were similar in PsO patients and controls (7.9 and 7.0%, respectively). However, the number of patients with PsA was low (23) and not sufficient to a reliable evaluation of AITD in these last patients (17).

Conversely, a subsequent study evaluated prospectively the prevalence of other autoimmune disorders in outpatient clinic in 3,069 consecutive patients with diagnosed chronic AT, with respect to two age- and sex-matched control groups: (a) a control group of 1,023 subjects, extracted from a random sample of the general population without thyroid disorders and (b) 1,023 patients with non-toxic multinodular goiter drawn by the same random sample of the general population, with similar iodine intake. The results of our study demonstrated a significant increase of the prevalence of PsA in AT patients (24).

A more recent study (18) aimed to assess the incidence of new cases of clinical and subclinical thyroid dysfunction (TD) in a broad group of PsA patients versus a control group, matched by age and gender with a similar iodine intake. PsA patients with TD were excluded first, and new cases of thyroid disorders were evaluated in 97 PsA patients and 97 matched controls (median follow-up of 74 months in PsA versus 92 in controls). A raised rate of new cases of hypothyroidism, TD, positive AbTPO, and appearance of a small hypoechoic thyroid pattern in PsA, especially in female gender, compared to controls has been evidenced. Risk factors in female gender for the development of TD were TSH within the normal range but at the higher limit, positive AbTPO, and small thyroid volume (18).

Interferon-γ and Th1 cytokines/chemokines are involved in the pathogenesis of PsO. Activated T cells and HLA-DR keratinocytes have been shown in active plaques (25). It has been shown that CXCL10, the Th1 prototype chemokine, and its receptor (CXCR)3 are present in keratinocytes and in the dermal infiltrate derived from active psoriatic plaques and that effective treatment of active plaques decreased the expression of CXCL10. Elevated circulating CXCL10 has been shown in PsO patients (25–28). The cellular infiltrate in acute plaques is constituted by 5–8% CD3(−)CD56(+) NK cells, as indicated by immunohistochemical techniques, especially localized in the mid and papillary dermis. NK lymphocyte migration toward CXCL10 is involved in the pathogenesis of PsO (27).

CXCL10 is a determinant chemoattractant for neutrophils, and an elevated infiltration and microabscess formation by neutrophils is a characteristic PsO feature. Different papers have shown a critical pathogenic role of neutrophils in PsO, particularly in the first phases, leading to hypothesize that blocking neutrophil function could have therapeutic effectiveness in this disease (23, 29, 30).

Also, in PsA patients, high levels of CXCL10 are observed in synovial fluid, and in circulation. Th1 cells immune predominance has been also shown at the beginning of the disease, with a subsequent later decline in long-lasting PsO or PsA, suggesting a shift from Th1 to Th2 immune response in long duration diseases (31–33).

Also, AITD are Th1 immune-mediated autoimmune disorders in which Th1 lymphocytes, IFN-γ, and IFN-γ dependent chemokines (CXCL9, CXCL10, CXCL11) play an important role (34, 35).

Serum levels of CXCL10 (the Th1 prototype chemokine) and CCL2 (the Th2 prototype chemokine) were measured in 37 patients with PsA without AT (PsA) and 28 with AT (PsA + AT), and in gender- and age-matched controls. The results of the study demonstrated higher circulating CXCL10 and CCL2 in PsA patients than in control subjects. Furthermore, serum CXCL10 (but not CCL2) levels in PsA patients were significantly higher in the presence of AT (36). These data suggested that a Th1 immune predominace, both in PsA such as in AT, might be the immunopathogenetic base of the association of these diseases.

## Conclusion

Psoriasis is associated with an increased risk of other autoimmune disorders like ulcerative colitis, Crohn’s disease, and celiac disease (37). Sporadic cases of association of PsO and AITD have been reported. However, two different recent studies did not find any association between PsO and AITD.

Psoriatic arthritis is a chronic inflammatory arthritis that occurs approximately in 30% of PsO patients. In patients with PsO and PsA, an association with AITD has been shown by most of the studies in adults, but not in the juvenile form. In PsA women and men, thyroid autoimmunity (positive AbTPO antibodies, hypoechoic thyroid pattern) and subclinical hypothyroidism were more prevalent than in the general population. An association has been shown also in patients with PsO, arthritis, and IBD who have more frequently AITD.

A Th1 immune predominance has been shown in early PsO, and PsA, such as in AT, with high circulating levels of the Th1 prototype chemokine CXCL10 overall in the presence of the association with AT. These data suggest that this Th1 immune predominance might be the immunopathogenetic base of the association of these disorders.

A very recent longitudinal study in PsA patients has shown a raised incidence of new cases of hypothyroidism, TD, positive AbTPO, and appearance of a small and hypoechoic thyroid in PsA, especially in female gender, compared to controls. Risk factors in female gender for the development of TD are TSH within the normal range but at the higher limit, positive AbTPO, and small thyroid volume, suggesting to evaluate AbTPO levels, thyroid function, and thyroid ultrasound, especially in PsA women. Thyroid function follow-up and suitable treatments should be performed regularly in PsA female patients at high risk TSH within the normal range but at the higher limit, positive AbTPO, hypoechoic, and small thyroid.

However, studies in larger number of patients are required to evaluate if routine thyroid screening could be beneficial for PsO patients.

## Author Contributions

IR, FR, SB, RV, AA, PF, and SMF gave substantial contribution in the conception and design of the work, and in writing the paper; gave the final approval of the version to be published; agreed to be accountable for all aspects of the work in ensuring that questions related to the accuracy or integrity of any part of the work are appropriately investigated and resolved. AA and SB revised it critically for important intellectual content.

## Conflict of Interest Statement

The authors declare that the research was conducted in the absence of any commercial or financial relationships that could be construed as a potential conflict of interest. The reviewer, MB, and handling editor declared their shared affiliation, and the handling editor states that the process nevertheless met the standards of a fair and objective review.

## Abbreviations

AITDs, autoimmune thyroid diseases; AT, autoimmune thyroiditis; AbTg, anti-thyroglobulin antibodies; AbTPO, antithyroid peroxidase antibodies; FT4, free thyroxine; IBD, inflammatory bowel disease; IFN, interferon; IL, interleukin; JIA, juvenile idiopathic arthritis; PsA, psoriatic arthritis; PsO, psoriasis; RA, rheumatoid arthritis; RF, rheumatoid factor; TD, thyroid dysfunction; TSH, thyroid-stimulating hormone.

## References

[1] Menter A Gottlieb A Feldman SR Van Voorhees AS Leonardi CL Gordon KB Guidelines of care for the management of psoriasis and psoriatic arthritis: Section 1. Overview of psoriasis and guidelines of care for the treatment of psoriasis with biologics. J Am Acad Dermatol (2008) 58:826–50. 10.1016/j.jaad.2008.02.039 18423260

[2] Parisi R Symmons DP Griffiths CE Ashcroft DM Identification and Management of Psoriasis and Associated ComorbidiTy (IMPACT) project team. Global epidemiology of psoriasis: a systematic review of incidence and prevalence. J Invest Dermatol (2013) 133:377–85. 10.1038/jid.2012.339 23014338

[3] Sima J Dermatology: Illustrated Study Guide and Comprehensive Board Review. New York: Springer International Publishing (2012).

[4] Ouyang W. Distinct roles of IL-22 in human psoriasis and inflammatory bowel disease. Cytokine Growth Factor Rev (2010) 21:435–41. 10.1016/j.cytogfr.2010.10.007 21106435

[5] Raychaudhuri SK Maverakis E Raychaudhuri SP. Diagnosis and classification of psoriasis. Autoimmun Rev (2014) 13:490–5. 10.1016/j.autrev.2014.01.008 24434359

[6] Parrish L. Psoriasis: symptoms, treatments and its impact on quality of life. Br J Community Nurs (2012) 17:524, 526, 528. 10.12968/bjcn.2012.17.11.524 23124421

[7] Palfreeman AC McNamee KE McCann FE. New developments in the management of psoriasis and psoriatic arthritis: a focus on apremilast. Drug Des Devel Ther (2013) 7:201–10. 10.2147/DDDT.S32713 23569359 PMC3615921

[8] Nestle FO Kaplan DH Barker J Psoriasis. N Engl J Med (2009) 361:496–509. 10.1056/NEJMra0804595 19641206

[9] Durham LE Taams LS Kirkham BW Psoriatic arthritis. Br J Hosp Med (Lond) (2016) 77:C102–8. 10.12968/hmed.2016.77.7.C102 27388392

[10] Goldenstein-Schainberg C Favarato MH Ranza R. Current and relevant concepts in psoriatic arthritis. Rev Bras Reumatol (2012) 52:98–106. 10.1590/S0482-50042012000100010 22286649

[11] Krawczyk-Wasielewska A Skorupska E Samborski W. Sacroiliac joint pain as an important element of psoriatic arthritis diagnosis. Postepy Dermatol Alergol (2013) 30:108–12. 10.5114/pdia.2013.34161 24278057 PMC3834688

[12] Antonelli A Delle Sedie A Fallahi P Ferrari SM Maccheroni M Ferrannini E High prevalence of thyroid autoimmunity and hypothyroidism in patients with psoriatic arthritis. J Rheumatol (2006) 33:2026–8. 17014017

[13] Molta CT Khan MA Aponte CJ Reynolds TL Macintyre SS. Familial occurrence of systemic sclerosis, rheumatoid arthritis and other immunological disorders: report of two kindreds with study of HLA antigens and review of the literature. Clin Exp Rheumatol (1989) 7:229–36. 2667829

[14] Nogita T Aramoto Y Terajima S Akimoto K Kawashima M Hidano A The coexistence of psoriasis vulgaris, Sjögren’s syndrome, and Hashimoto’s thyroiditis. J Dermatol (1992) 19:302–5. 10.1111/j.1346-8138.1992.tb03229.x 1644955

[15] Bianchi G Marchesini G Zoli M Falasconi MC Iervese T Vecchi F Thyroid involvement in chronic inflammatory rheumatological disorders. Clin Rheumatol (1993) 12:479–84. 10.1007/BF02231775 8124909

[16] Gul U Gonul M Kaya I Aslan E Autoimmune thyroid disorders in patients with psoriasis. Eur J Dermatol (2009) 9:221–3. 10.1684/ejd.2009.0632 19251564

[17] Vassilatou E Papadavid E Papastamatakis P Alexakos D Koumaki D Katsimbri P No association of psoriasis with autoimmune thyroiditis. J Eur Acad Dermatol Venereol (2017) 31:102–6. 10.1111/jdv.13767 27324349

[18] Fallahi P Ferrari SM Ruffilli I Elia G Miccoli M Sedie AD Increased incidence of autoimmune thyroid disorders in patients with psoriatic arthritis: a longitudinal follow-up study. Immunol Res (2017) 65:681–6. 10.1007/s12026-017-8900-8 28101810

[19] Soy M Guldiken S Arikan E Altun BU Tugrul A. Frequency of rheumatic diseases in patients with autoimmune thyroid disease. Rheumatol Int (2007) 27:575–7. 10.1007/s00296-006-0263-8 17102943

[20] Unsal E Oren O Salar K Makay B Abaci A Ozhan B The frequency of autoimmune thyroid disorders in juvenile idiopathic arthritis. Turk J Pediatr (2008) 50:462–5. 19102051

[21] Binus AM Han J Qamar AA Mody EA Holt EW Qureshi AA. Associated comorbidities in psoriasis and inflammatory bowel disease. J Eur Acad Dermatol Venereol (2012) 26:644–50. 10.1111/j.1468-3083.2011.04153.x 21689167

[22] Sardu C Cocco E Mereu A Massa R Cuccu A Marrosu MG Population based study of 12 autoimmune diseases in Sardinia, Italy: prevalence and comorbidity. PLoS One (2012) 7:e32487. 10.1371/journal.pone.0032487 22396771 PMC3292563

[23] Ferrari SM Ruffilli I Colaci M Antonelli A Ferri C Fallahi P. CXCL10 in psoriasis. Adv Med Sci (2015) 60:349–54. 10.1016/j.advms.2015.07.011 26318079

[24] Fallahi P Ferrari SM Ruffilli I Elia G Biricotti M Vita R The association of other autoimmune diseases in patients with autoimmune thyroiditis: review of the literature and report of a large series of patients. Autoimmun Rev (2016) 15:1125–8. 10.1016/j.autrev.2016.09.009 27639841

[25] Gottlieb AB Luster AD Posnett DN Carter DM. Detection of a gamma interferon-induced protein IP-10 in psoriatic plaques. J Exp Med (1988) 168:941–8. 10.1084/jem.168.3.941 2459293 PMC2189020

[26] Boorsma DM Flier J Sampat S Ottevanger C de Haan P Hooft L Chemokine IP-10 expression in cultured human keratinocytes. Arch Dermatol Res (1998) 290:335–41. 10.1007/s004030050314 9705166

[27] Ottaviani C Nasorri F Bedini C de Pità O Girolomoni G Cavani A. CD56brightCD16(-) NK cells accumulate in psoriatic skin in response to CXCL10 and CCL5 and exacerbate skin inflammation. Eur J Immunol (2006) 36:118–28. 10.1002/eji.200535243 16323244

[28] Deeva I Mariani S De Luca C Pacifico V Leoni L Raskovic D Wide-spectrum profile of inflammatory mediators in the plasma and scales of patients with psoriatic disease. Cytokine (2010) 49:163–70. 10.1016/j.cyto.2009.09.014 19879157

[29] Naik HB Cowen EW. Autoinflammatory pustular neutrophilic diseases. Dermatol Clin (2013) 31:405–25. 10.1016/j.det.2013.04.001 23827244 PMC3703099

[30] Christophers E Metzler G Röcken M. Bimodal immune activation in psoriasis. Br J Dermatol (2014) 170:59–65. 10.1111/bjd.12631 24117368

[31] Proost P Struyf S Loos T Gouwy M Schutyser E Conings R Coexpression and interaction of CXCL10 and CD26 in mesenchymal cells by synergising inflammatory cytokines: CXCL8 and CXCL10 are discriminative markers for autoimmune arthropathies. Arthritis Res Ther (2006) 8:R107. 10.1186/ar1997 16846531 PMC1779382

[32] Lande R Giacomini E Serafini B Rosicarelli B Sebastiani GD Minisola G Characterization and recruitment of plasmacytoid dendritic cells in synovial fluid and tissue of patients with chronic inflammatory arthritis. J Immunol (2004) 173:2815–24. 10.4049/jimmunol.173.4.2815 15295000

[33] Antonelli A Fallahi P Delle Sedie A Ferrari SM Maccheroni M Bombardieri S High values of Th1 (CXCL10) and Th2 (CCL2) chemokines in patients with psoriatic arthritis. Clin Exp Rheumatol (2009) 27:22–7. 19327225

[34] Antonelli A Ferrari SM Corrado A Di Domenicantonio A Fallahi P. Autoimmune thyroid disorders. Autoimmun Rev (2015) 14:174–80. 10.1016/j.autrev.2014.10.016 25461470

[35] Antonelli A Ferrari SM Frascerra S Pupilli C Mancusi C Metelli MR CXCL9 and CXCL11 chemokines modulation by peroxisome proliferator-activated receptor-alpha agonists secretion in Graves’ and normal thyrocytes. J Clin Endocrinol Metab (2010) 95:E413–20. 10.1210/jc.2010-0923 20810571

[36] Antonelli A Fallahi P Delle Sedie A Ferrari SM Maccheroni M Bombardieri S High values of alpha (CXCL10) and beta (CCL2) circulating chemokines in patients with psoriatic arthritis, in presence or absence of autoimmune thyroiditis. Autoimmunity (2008) 41:537–42. 10.1080/08916930802170401 18855195

[37] Bhatia BK Millsop JW Debbaneh M Koo J Linos E Liao W. Diet and psoriasis, part II: celiac disease and role of a gluten-free diet. J Am Acad Dermatol (2014) 71:350–8. 10.1016/j.jaad.2014.03.017 24780176 PMC4104239

